# Adherence to the 2012 American College of Rheumatology (ACR) Guidelines for Management of Gout: A Survey of Brazilian Rheumatologists

**DOI:** 10.1371/journal.pone.0135805

**Published:** 2015-08-14

**Authors:** Ana Beatriz Vargas-Santos, Geraldo da Rocha Castelar-Pinheiro, Evandro Silva Freire Coutinho, H. Ralph Schumacher, Jasvinder A. Singh, Naomi Schlesinger

**Affiliations:** 1 Division of Rheumatology, Internal Medicine Department, Universidade do Estado do Rio de Janeiro, Rio de Janeiro, Rio de Janeiro, Brazil; 2 Epidemiology Department, Escola Nacional de Saúde Pública Sergio Arouca, Fundação Oswaldo Cruz, Rio de Janeiro, Rio de Janeiro, Brazil; 3 Division of Rheumatology, University of Pennsylvania School of Medicine and Veterans Affairs Medical Center, Philadelphia, Pennsylvania, United States of America; 4 Medicine Service, Birmingham Veterans Affairs Medical Center, Birmingham, Alabama, United States of America; 5 Department of Medicine at the School of Medicine, and the Division of Epidemiology at School of Public Health, University of Alabama, Birmingham, Alabama, United States of America; 6 Department of Orthopedic Surgery, Mayo Clinic College of Medicine, Rochester, Minnesota, United States of America; 7 Division of Rheumatology, Department of Medicine, Rutgers–Robert Wood Johnson Medical School, New Brunswick, New Jersey, United States of America; David Geffen School of Medicine, UNITED STATES

## Abstract

**Objective:**

To describe the current pharmacological approach to gout treatment reported by rheumatologists in Brazil.

**Methods:**

We performed a cross-sectional survey study using an online questionnaire e-mailed to 395 rheumatologists, randomly selected, from among the members of the Brazilian Society of Rheumatology.

**Results:**

Three hundred and nine rheumatologists (78.2%) responded to the survey. For acute gout attacks, combination therapy (NSAIDs or steroid + colchicine) was often used, even in monoarticular involvement, and colchicine was commonly started as monotherapy after 36 hours or more from onset of attack. During an acute attack, urate-lowering therapy (ULT) was withdrawn by approximately a third of rheumatologists. Anti-inflammatory prophylaxis (98% colchicine) was initiated when ULT was started in most cases (92.4%), but its duration was varied. Most (70%) respondents considered the target serum uric acid level to be less than 6 mg/dl. Approximately 50% of rheumatologists reported starting allopurinol at doses of 100 mg daily or less and 42% reported the initial dose to be 300 mg daily in patients with normal renal function. ULT was maintained indefinitely in 76% of gout patients with tophi whereas in gout patients without tophi its use was kept indefinitely in 39.6%.

**Conclusion:**

This is the first study evaluating gout treatment in a representative, random sample of Brazilian rheumatologists describing common treatment practices among these specialists. We identified several gaps in reported gout management, mainly concerning the use of colchicine and ULT and the duration of anti-inflammatory prophylaxis and ULT. Since rheumatologists are considered as opinion leaders in this disease, a program for improving quality of care for gout patients should focus on increasing their knowledge in this common disease.

## Introduction

Gout is the most common inflammatory arthritis and one of the oldest known arthritides. Its prevalence has increased for the last few decades [1–4]. It is one of the few rheumatic diseases where etiology, pathophysiology, diagnosis and treatment are well established. Despite the existence of this knowledge, gout is often poorly managed [5–11].

This paradox has been investigated. Some potential explanations were: 1) doctors’ misconception that gout is a well-known and perhaps “unimportant disease”, so there is no need for regular scientific updating and 2) patients’ lack of information about their disease and its management which may partially explain their poor adherence to treatment [12–15]. On the other hand, some changes in the last decade may have impacted the current scenario. There is a growing interest in gout as documented by the increasing number of publications in this field, including the launch of new drugs for the treatment of gout and studies related to increasing gout prevalence and the poor quality of care of gout patients [1–13,16,17]. There is a need to translate this knowledge into better practice. Several national and international organizations compiled the available evidence into management guidelines and recommendations since 2006 [18–25]. A major challenge to these guidelines relates to their effective adoption by the practicing health care providers. Adherence to guidelines needs to be assessed in order to improve the quality of care provided to gout patients.

Many studies have evaluated doctors’ reports of their approaches to treating patients with gout [26–44] (S1 Table). With few exceptions, response rates in these studies have varied from 21% to 58%, thus, limiting their generalizability. Evidence-based guidelines for treatment of gout were published by the American College of Rheumatology (ACR) in 2012 [22,23]. Adherence to these guidelines has already been evaluated [45], but, to our knowledge, ours is the first survey to do it in a representative national sample of rheumatologists. The objectives of our study were to evaluate the current pharmacological treatment of gout as reported by rheumatologists in Brazil, and to assess where gaps in recommended care exist, so that future continued medical education programs can target these gaps and address them in a comprehensive fashion.

## Materials and Methods

### Study design and population

A cross-sectional study was undertaken in a population of practicing rheumatologists, members of the Brazilian Society of Rheumatology (BSR).

### Sample design

A simple random sample was obtained from a list of 1436 practicing rheumatologists provided by the BSR, which was made available to us for this study. The sample was stratified proportionally to the number of rheumatologists in each Brazilian geographic region to prevent the absence of rheumatologists from areas with a small number of specialists. The sample size was calculated according to the following parameters: a confidence level of 95% and an error of 5%, considering an expected proportion of correct answers of 50% for the most important question in the authors’ opinion (target serum uric acid [SUA] level). Using PEPI (Programs for Epidemiologists, version 11.39), the sample size was calculated as 304 individuals. Considering a possible failure to contact 20–30% of the sample, 395 members were randomly selected using the online tool “True Random Number Generator” available in www.random.org. The study was approved by the Ethics Committee of Hospital Universitário Pedro Ernesto. The Ethics Committee waived the requirement to obtain a signed consent form. The submission of the completed questionnaire was deemed to constitute consent to participate. All data were analyzed anonymously.

### Data collection

A self-administered questionnaire was developed using the online survey tool SurveyMonkey (SurveyMonkey, Palo Alto, California, USA– www.surveymonkey.com). The survey was available from December 18, 2013 to March 25, 2014. During that period, up to four invitations were sent via e-mail and up to three telephone calls were made for each potential participant. The questionnaire is attached as a supplement (S1 File).

### Domains assessed

The survey included questions regarding the treatment of acute attack as well as treatment with the urate-lowering therapy (ULT) and prophylaxis. It also included questions about demographics and training/experience in rheumatology, number of gout patients seen monthly and practice setting (private *versus* [*vs*.] academic *vs*. both). The first set of questions was regarding the preferred drug to treat an acute gout attack in eight different scenarios and responses included colchicine (high dose, low dose), nonsteroidal anti-inflammatory drugs (NSAIDs), corticosteroids (oral, intramuscular, intraarticular), or combinations of two of these drugs. These scenarios included different combinations of three features: the number of affected joints (mono *vs*. polyarticular), duration of the acute attack at the time the treatment was to be started (<36 hours *vs*. >36 hours), and the presence of chronic kidney disease (CKD) (healthy patient *vs*. patient with a creatinine clearance [CrCl] less than 60 ml/min).

The next set of questions queried the management of the ULT during an acute gout attack (continuation, discontinuation, dose modification); anti-inflammatory prophylaxis to prevent gout attacks during ULT initiation (colchicine *vs*. NSAIDs; duration); indications, starting dose and duration of ULT after achieving the target SUA level (discontinuation, 1–6 months, 7–11 months, 1–3 years, ≥4 years, until tophi resolution, indefinite); target SUA level (<6 *vs*. <5 *vs*. <6.8 mg/dl *vs*. <upper limit of normal *vs*. no consideration for SUA level), and frequency of laboratory evaluation (until the target SUA achieved and after that; every 1–3 months, every 4–6 months, every 7–9 months, every 10–12 months). Probenecid, febuxostat and pegloticase were not included in the survey because they are not available in Brazil.

### Outcome: Concordance with 2012 ACR gout guidelines

We assessed the concordance of rheumatologist-reported practices with the following 2012 ACR gout guidelines: 1) “For countries where 1.0 mg or 0.5 mg rather than 0.6 mg tablets of colchicine are available, the task force panel (TFP) recommended, as appropriate, 1.0 mg colchicine as the loading dose, followed by 0.5 mg 1 hour later, and then followed, as needed, after 12 hours, by continued colchicine (up to 0.5 mg 3 times daily) until the acute attack resolves”; 2) “Colchicine was recommended as an appropriate option for acute gout if started within 36 hours of symptom onset”; 3) “Ongoing pharmacologic ULT should not be interrupted during an acute gout attack”; 4) “The minimum serum urate target is <6 mg/dl. Serum urate lowering below 5 mg/dl may be needed to improve gout signs and symptoms”; 5) Allopurinol–“Starting dosage should be no greater than 100 mg/day for any patient, and start at 50 mg/day in stage 4 or worse CKD”.

The specific outcomes assessed related to the management of acute gout and ULT were: 1) no prescription of high dose colchicine, i.e., 0.5 mg/hour until symptom resolution or side effect, to treat acute gout; 2) no prescription of colchicine as monotherapy to treat acute attacks lasting more than 36 hours; 3) maintenance of ULT dose during acute gout attack; 4) target SUA level <6 or <5 mg/dl for patients with tophi and for patients with no tophi; 5) initial dose of allopurinol of 50 mg/day or 100 mg/day for patients with normal renal function and for patients with a CrCl ≤60 ml/min.

### Statistical analysis

We calculated proportions and their 95% confidence intervals (95% CIs) for categorical variables and means and standard deviations for continuous variables. The statistical significance of the differences between respondents and non-respondents was assessed by chi-squared test or Student’s t-test, as appropriate.

Proportions of rheumatologists’ responses on managing acute gout and ULT management in acute and chronic gout were compared. We evaluated the potential predictors of concordance of rheumatologist’s practice with the ACR guidelines. Seven dependent variables mentioned above were assessed. Firstly, univariate logistic regression models were fitted for the following ten independent variables related to the rheumatologist: gender, age, time since medical school graduation, years of practicing rheumatology, geographic region, academic activity, residency in rheumatology, attending the 2013 BSR Meeting, attending any conference on gout in the 2013 BSR Meeting and average number of patients with gout seen in one month. Variables with p-values ≤0.20 were selected for subsequent multivariate analysis [46] and those with p-value ≤0.10 were kept in the final model.

All p-values were two-sided. P-values ≤0.05 and between 0.06 and 0.10 were considered statistically significant and of borderline significance, respectively. Stata software (version 12.0, College Station, TX, USA) was used for all statistical analysis.

## Results

**Fig 1:**
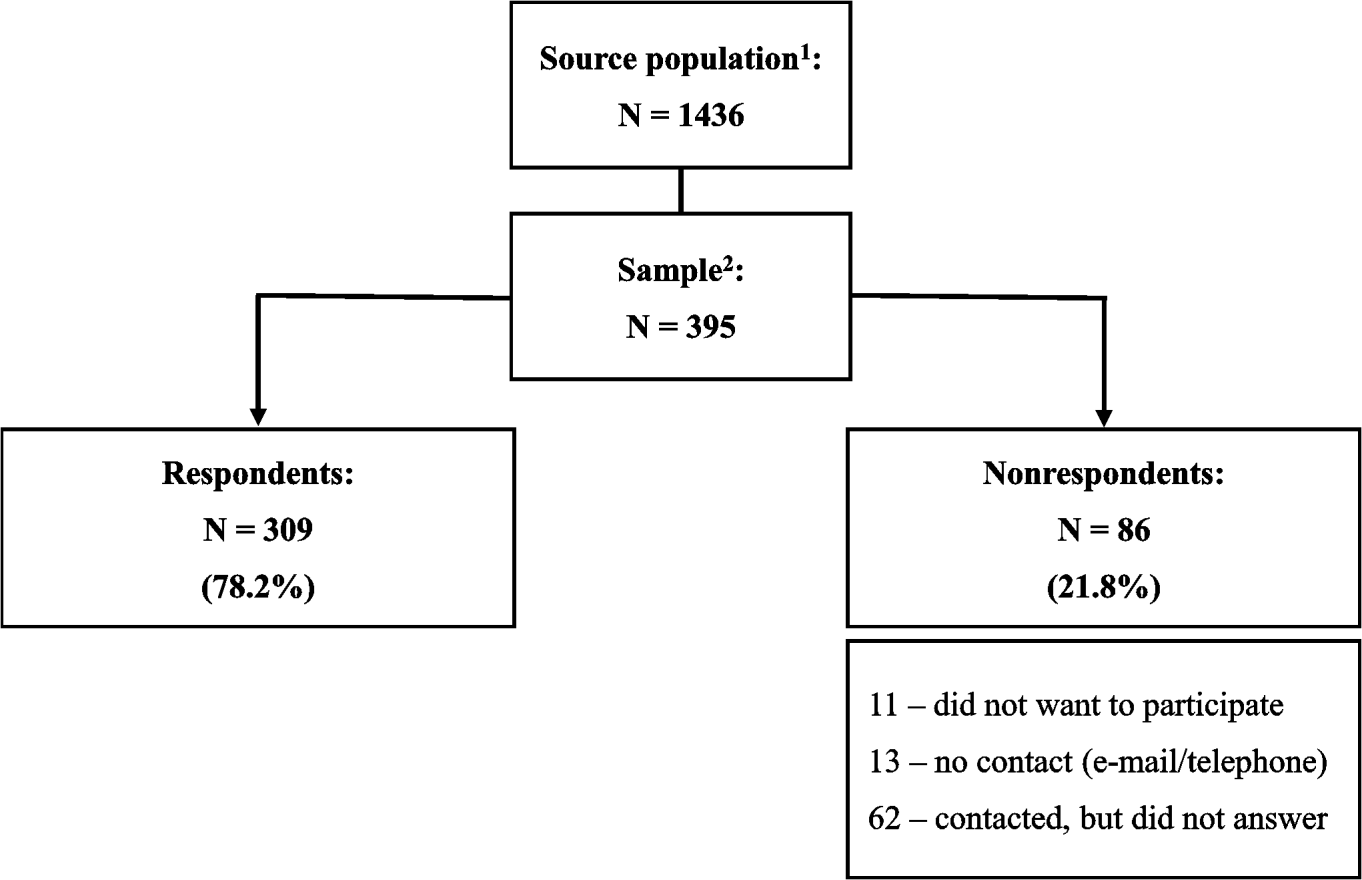
Flow diagram of participants’ selection. 1: Practicing rheumatologists, members of the Brazilian Society of Rheumatology; 2: Random sample, stratified proportionally to the number of rheumatologists in each Brazilian geographic region.

Of the original sample of 395 rheumatologists, 309 (78.2%) responded to the survey. Fig 1 shows the flow diagram of participants’ selection.

Demographic and other characteristics of the sample are presented in Table 1. The mean age of the participants was 46.1 years (range: 29–88 years, median 44 years), 58% were women, and approximately 2/3^rd^ had >10 years of experience in rheumatology and provided care to ≤10 gout patients per month. Fifty three percent were only in private practice and 87% were board certified by the BSR.

**Table 1:** Description of the study sample and comparison between respondents and nonrespondents.

Feature	Respondents	Nonrespondents	P value
**Age** ^1^	**[N = 309]**	**[N = 78]** ^2^	
	46.1 years (±12.0)	51.6 years (±12.5)	<0.001
**Female gender** ^**3**^	**[N = 309]**	**[N = 86]**	
	58.3 (52.7–63.8)	46.5 (35.8–57.3)	0.08
**Time practicing rheumatology** ^**3**^	**[N = 302]**	**N/A**	
0–10 years	37.4 (31.9–42.9)	N/A	
11–20 years	22.5 (17.8–27.3)	N/A	
21–30 years	22.2 (17.5–26.9)	N/A	
≥31 years	17.9 (13.5–22.2)	N/A	
**Gout patients seen monthly** ^**3**^	**[N = 302]**	**N/A**	
0–5 patients	33.1 (27.8–38.5)	N/A	
6–10 patients	35.8 (30.3–41.2)	N/A	
11–20 patients	20.9 (16.3–25.5)	N/A	
≥21 patients	10.3 (6.8–13.7)	N/A	
**Type of practice** ^**3**^	**[N = 302]**	**N/A**	
Academic	1.3 (0.0–2.6)	N/A	
Private	53.3 (47.7–59.0)	N/A	
Combined	45.4 (39.7–51.0)	N/A	
**Board certified (BSR)** ^**3**^	**[N = 309]**	**[N = 86]**	
Yes	86.7 (82.9–90.5)	70.9 (61.1–80.7)	0.001
**2013 BSR Meeting** ^**3**^	**[N = 309]**	**[N = 86]**	
Yes	64.1 (58.7–69.5)	46.5 (35.8–57.3)	0.003

^1^Mean (standard deviation)

^2^8 missing data

^**3**^proportion (95% confident interval).

N/A: not available; BSR: Brazilian Society of Rheumatology.

Compared to survey responders, nonresponders were older (51.6 *vs*. 46.1 years, p <0.001), more likely to be men (53 *vs*. 42%, p = 0.08), fewer were board certified rheumatologists (71 *vs*. 87%, p = 0.001) and fewer attended the 2013 BSR Meeting (47 *vs*. 64%, p = 0.003).

### Acute gout treatment

A combination of an NSAID plus colchicine was the preferred treatment option for an acute gout attack in an otherwise healthy patient, ranging from 38.5% (polyarticular, >36 hours) to 51.5% (polyarticular, <36 hours), while the combination of a corticosteroid with colchicine was the most common choice for patients with CKD, from 27.5% (monoarticular, <36 hours) to 47.6% (polyarticular, >36 hours) (Table 2).

**Table 2:** Acute gout management: first choice drug(s) to treat an acute gouty attack in different scenarios (N = 309).

Gout attack	Monoarticular				Polyarticular			
Symptoms’ onset	<36 hours		>36 hours		<36 hours		>36 hours	
Health status	Healthy^1^	CKD^2^	Healthy	CKD	Healthy	CKD	Healthy	CKD
Scenario	1	2	3	4	5	6	7	8
Medication	% (CI 95%)	% (CI 95%)	% (CI 95%)	% (CI 95%)	% (CI 95%)	% (CI 95%)	% (CI 95%)	% (CI 95%)
High colchicine^3^	5.2 (2.7–7.7)	2.9 (1.0–4.8)	1.6 (0.2–3.0)	2.3 (0.6–3.9)	2.3 (0.6–3.9)	1.9 (0.4–3.5)	0.0	1.3 (0.0–2.6)
Low colchicine^4^	16.5 (12.3–20.7)	23.0 (18.3–27.7)	6.8 (4.0–9.6)	12.3 (8.6–16.0)	3.2 (1.3–5.2)	8.7 (5.6–11.9)	3.2 (1.3–5.2)	6.5 (3.7–9.2)
NSAID	22.3 (17.7–27.0)	1.0 (0.0–2.1)	22.3 (17.7–27.0)	1.9 (0.4–3.5)	15.5 (11.5–19.6)	1.0 (0.0–2.1)	13.9 (10.0–17.8)	1.0 (0.0–2.1)
PO steroid	0.3 (0.0–1.0)	15.9 (11.8–20.0)	0.6 (0.0–1.5)	18.8 (14.4–23.1)	2.3 (0.6–3.9)	25.2 (20.4–30.1)	2.9 (1.0–4.8)	25.9 (21.0–30.8)
IM steroid	1.0 (0.0–2.1)	7.8 (4.8–10.8)	1.9 (0.4–3.5)	8.1 (5.0–11.1)	3.6 (1.5–5.6)	14.6 (10.6–18.5)	3.6 (1.5–5.6)	14.2 (10.3–18.2)
IA steroid	2.3 (0.6–3.9)	18.1 (13.8–22.4)	3.9 (1.7–6.0)	17.5 (13.2–21.7)	0.0	0.3 (0.0–1.0)	0.0	1.0 (0.0–2.1)
NSAID + colchicine	46.3 (40.7–51.9)	2.6 (0.8–4.4)	46.9 (41.3–52.5)	2.3 (0.66–3.9)	51.5 (45.9–57.1)	1.0 (0.0–2.1)	38.5 (33.1–44.0)	1.0 (0.0–2.1)
NSAID + steroid (PO/IM/IA)	1.9 (0.4–3.5)	1.3 (0.0–2.6)	6.8 (4.0–9.6)	1.3 (0.0–2.6)	6.8 (4.0–9.6)	1.6 (0.2–3.0)	14.2 (10.3–18.2)	1.6 (0.2–3.0)
Steroid (PO/IM/IA) + colchicine	4.2 (2.0–6.5)	27.5 (22.5–32.5)	9.1 (5.8–12.3)	35.6 (30.2–41.0)	14.9 (10.9–18.9)	45.6 (40.0–51.2)	23.6 (18.9–28.4)	47.6 (42.0–53.2)

^1^Healthy besides gout

^2^chronic kidney disease, defined here as creatinine clearance ≤60 ml/min

^3^colchicine 0.5 mg/hour until symptom resolution or side effect

^4^colchicine ≤2 mg/day. CI: confidence interval

NSAID: nonsteroidal anti-inflammatory drug; PO: *per os*; IM: intramuscular; IA: intra-articular.

More than 90% of respondents reported to always or almost always prescribe a prophylactic treatment when initiating ULT (Table 3), in concordance with the ACR guidelines.

**Table 3:** Management of urate-lowering therapy (ULT) during an acute gouty attack and anti-inflammatory prophylaxis of gout attacks.

Questions and respective options	Proportion (95% CI)
**During an acute gouty attack in a patient using ULT, you:**	**[N = 309]**
Increase the ULT dose	0.3 (0.0–1.0)
Keep the ULT dose^1^	67.0 (61.7–72.3)
Reduce the ULT dose	1.3 (0.0–2.6)
Withdraw the ULT	31.4 (26.2–36.6)
**How often do you give prophylactic treatment to prevent acute gouty attacks when initiating ULT?**	**[N = 302]**
Always^1^	64.9 (59.5–70.3)
Almost always^1^	27.5 (22.4–32.5)
Sometimes	3.6 (1.5–5.8)
Almost never	1.7 (0.2–3.1)
Never^2^	2.3 (0.6–4.0)
**How long do you keep prophylaxis for patients WITHOUT tophi?**	**[N = 295]**
<1 month	16.9 (12.6–21.3)
1–6 months	42.4 (36.7–48.0)
7–12 months	10.8 (7.3–14.4)
Until they reach the target serum uric acid level	20.0 (15.4–24.6)
Indefinitely	9.8 (6.4–13.2)
**How long do you keep prophylaxis for patients WITH tophi?**	**[N = 295]**
<1 month	5.1 (2.6–7.6)
1–6 months	23.4 (18.5–28.2)
7–12 months	15.6 (11.4–19.8)
Until they reach the target serum uric acid level	13.9 (9.9–17.9)
Until resolution of tophi	7.5 (4.4–10.5)
Indefinitely	34.6 (29.1–40.0)
**Do you prefer colchicine or NSAID for chronic prophylaxis of acute gouty attacks?**	**[N = 295]**
Colchicine	97.6 (95.9–99.4)
NSAID	2.4 (0.6–4.1)

^1^Answers in agreement with the 2012 ACR gout guidelines

^2^participants who answered not to prescribe anti-inflammatory prophylaxis when initiating ULT were excluded from the other questions concerning this topic.

CI: confidence interval; NSAID: nonsteroidal anti-inflammatory drug.

Analyzing the eight possible scenarios (Table 2) simultaneously, we identified that a significant proportion of rheumatologists indicated, once at least, the use of treatment modalities that are recommended not to be used as per the ACR guidelines: the use of high dose colchicine by 10% (95% CI, 6.7–13.4), and starting colchicine monotherapy more than 36 hours after the onset of the attack by 20.1% (95% CI, 15.6–24.6). During an acute attack, 31.4% of respondents withdrew ULT. There was great variation in the duration of anti-inflammatory prophylaxis as reported by the respondents: less than one month by 16.9% and 5.1% and indefinitely by 9.8% and 34.6% of respondents for patients without and with tophi, respectively (Table 3).

Table 4 shows the final multivariate models evaluating predisposing factors to answers about acute gout in concordance to the ACR guidelines. Considering that ORs lower than 1 represent a reduced chance of concordance with the ACR guidelines, the geographical regions showed a heterogeneous pattern of agreement for the three therapeutic approaches evaluated. Older professionals and those without academic practice were less likely to choose the recommended procedure in two out of the three provided situations. Finally, male rheumatologists had a 70% less chance of not prescribing high dose colchicine for acute gout.

**Table 4:** Multivariate-adjusted predictors of concordance between reported acute gout management and the 2012 ACR gout guidelines.

Outcomes [Pseudo R2]	OR (CI 95%)	P
**No prescription of high dose colchicine** ^1^ **for acute gout [0.1084]**		
a) Gender (ref.: female)		
Male	0.30 (0.13–0.68)	**0.004**
b) Activity (ref.: academic, with or without private)		
Private only	0.32 (0.14–0.77)	**0.011**
c) Geographic region of Brazil (ref.: South)		
Midwest	0.75 (0.14–4.14)	0.740
North	0.79 (0.07–8.79)	0.850
Northeast	0.29 (0.07–1.16)	0.080
Southeast	0.81 (0.22–3.05)	0.761
**No prescription of colchicine as monotherapy for acute gouty attack lasting >36 hours [0.0548]**		
a) Age (continuous variable in years)	0.97 (0.94–0.99)	**0.004**
b) Geographic region of Brazil (ref.: Northeast)		
Midwest	0.15 (0.04–0.55)	**0.005**
North	0.45 (0.07–2.86)	0.400
South	0.32 (0.09–1.14)	0.080
Southeast	0.27 (0.09–0.82)	**0.020**
**To keep ULT dose during an acute gouty attack [0.1496]**	** **	** **
a) Age (continuous variable in years)	0.92 (0.90–0.95)	**<0.001**
b) Activity (ref.: academic, with or without private)		
Private only	0.62 (0.36–1.07)	0.088
c) Geographic region of Brazil (ref.: South)		
Midwest	0.24 (0.08–0.73)	**0.012**
North	0.28 (0.07–1.20)	0.086
Northeast	0.55 (0.20–1.51)	0.243
Southeast	0.51 (0.22–1.20)	0.121

^1^Colchicine 0.5 mg/hour until symptom resolution or side effect.

ACR: American College of Rheumatology; OR: odds ratio; CI: confidence interval; ULT: urate-lowering therapy. OR lower than 1 represents a reduced chance of concordance with the 2012 ACR gout guidelines.

### Urate-lowering therapy

Table 5 summarizes the questions regarding ULT.

**Table 5:** Urate-lowering therapy (ULT).

Questions and respective options	Proportion (95% CI)
**When would you initiate ULT? Check all that apply.**	**[N = 308]**
After their first gouty attack	45.8 (40.2–51.4)
After two or more gouty attacks a year^1^	64.0 (58.6–69.4)
When a patient has tophi^1^	46.8 (41.1–52.4)
When a gout patient has CKD^1^	24.7 (19.8–29.5)
None of the above	3.6 (1.5–5.7)
**Choose the SUA level you consider the goal for ULT for a patient WITH TOPHI:** ^2^	**[N = 308]**
<5.0 mg/dl^1^	38.0 (32.5–43.4)
<6.0 mg/dl^1^	40.9 (35.4–46.4)
<6.8 mg/dl	5.5 (3.0–8.1)
<upper limit provided by the lab	7.8 (4.8–10.8)
I don’t adjust the ULT based on a specific level of serum uric acid.	7.8 (4.8–10.8)
**Choose the SUA level you consider the goal for ULT for a patient WITHOUT TOPHI:**	**[N = 283]**
<5.0 mg/dl^1^	15.5 (11.3–19.8)
<6.0 mg/dl^1^	54.4 (48.6–60.3)
<6.8 mg/dl	18.4 (13.8–22.9)
<upper limit provided by the lab	9.5 (6.1–13.0)
I don’t adjust the ULT based on a specific level of serum uric acid.	2.1 (0.4–3.8)
**If you were to initiate ULT in a patient, would you wait for the resolution of the acute gouty attack?**	**[N = 302]**
Yes	96.7 (94.7–98.7)
**How long after resolution of the acute gouty attack would you wait to initiate ULT?** ^**3**^	**[N = 292]**
1–3 weeks	76.7 (71.8–81.6)
4–6 weeks	21.6 (16.8–26.3)
7–9 weeks	0.7 (0.0–1.6)
10–12 weeks	1.0 (0.0–2.2)
**In general, when you initiate allopurinol, what is the initial dose you prescribe for a patient with normal renal function?**	**[N = 302]**
50 mg/day^1^	2.3 (0.6–4.0)
100 mg/day^1^	49.0 (43.3–54.7)
200 mg/day	6.0 (3.3–8.6)
300 mg/day	42.7 (37.1–48.3)
**In general, when you initiate allopurinol, what is the initial dose you prescribe for a patient with CrCl ≤60 ml/min?**	**[N = 299]**
50 mg/day^1^	28.4 (23.3–33.6)
100 mg/day^1^	62.5 (57.0–68.1)
200 mg/day	5.0 (2.5–7.5)
300 mg/day	4.0 (1.8–6.3)
**Do you prescribe benzbromarone for patients with CrCl >60 ml/min?**	**[N = 302]**
Always	7.9 (4.9–11.0)
Almost always	16.6 (12.3–20.8)
Sometimes	47.0 (41.4–52.7)
Almost never	14.2 (10.3–18.2)
Never	14.2 (10.3–18.2)
**Do you prescribe benzbromarone for patients with CrCl between 30 and 60 ml/min?**	**[N = 302]**
Always	3.3 (1.3–5.3)
Almost always	7.6 (4.6–10.6)
Sometimes	22.2 (17.5–26.9)
Almost never	35.4 (30.0–40.9)
Never	31.5 (26.2–36.7)
**Do you prescribe benzbromarone for patients with CrCl <30 ml/min?**	**[N = 302]**
Always	2.0 (0.4–3.6)
Almost always	2.6 (0.8–4.5)
Sometimes	9.3 (6.0–12.6)
Almost never	15.6 (11.5–19.7)
Never	70.5 (65.4–75.7)
**Do you prescribe benzbromarone for patients with current renal underexcretion of uric acid and a history of kidney stones in the past?**	**[N = 302]**
Always	3.6 (1.5–5.8)
Almost always	5.0 (2.5–7.4)
Sometimes	12.9 (9.1–16.7)
Almost never	20.9 (16.3–25.5)
Never	57.6 (52.0–63.2)
**Do you prescribe benzbromarone for patients also using allopurinol?**	**[N = 302]**
Always	1.7 (0.2–3.1)
Almost always	6.3 (3.5–9.0)
Sometimes	42.1 (36.5–47.7)
Almost never	21.5 (16.9–26.2)
Never	28.5 (23.4–33.6)
**For how long after achieving the target SUA level do you prescribe ULT for a patient with gout WITHOUT tophi?**	**[N = 283]**
Withdraw the medication	2.1 (0.4–3.8)
Maintain for 1–6 months	21.6 (16.7–26.4)
Maintain for 7–11 months	11.7 (7.9–15.4)
Maintain for 1–3 years	17.3 (12.9–21.7)
Maintain for 4 or more years	7.8 (4.6–10.9)
Maintain indefinitely^1^	39.6 (33.8–45.3)
**For how long after achieving the target SUA level do you prescribe ULT for a patient with gout WITH tophi?**	**[N = 283]**
Withdraw the medication	1.4 (0.0–2.8)
Maintain for 1–6 months	2.8 (0.9–4.8)
Maintain for 7–11 months	4.2 (1.9–6.6)
Maintain for 1–3 years	6.4 (3.5–9.2)
Maintain for 4 or more years	3.2 (1.1–5.2)
Maintain until tophi resolution	5.7 (2.9–8.4)
Maintain indefinitely^1^	76.3 (71.3–81.3)
**How often do you check your patients SUA levels BEFORE achieving uric-acid levels goals?**	**[N = 283]**
Every 1–3 months^1^	80.6 (75.9–85.2)
Every 4–6 months	19.1 (14.5–23.7)
Every 7–9 months	0.4 (0.3–1.0)
Every 10–12 months	0.0 (-)
**How often do you check your patients SUA levels AFTER achieving uric-acid levels goals?**	**[N = 283]**
Every 1–3 months	5.7 (2.9–8.4)
Every 4–6 months^1^	71.4 (66.1–76.7)
Every 7–9 months	13.1 (9.1–17.0)
Every 10–12 months	9.9 (6.4–13.4)

^1^Answers in agreement with the 2012 American College of Rheumatology gout guidelines

^2^participants who reported not to adjust ULT for patients with tophaceous gout based on the SUA level were excluded from other questions that were based on the concept of a target SUA level

^3^this question was offered only to those who answered that they would wait for the resolution of the acute gouty attack to initiate ULT.

CI: confidence interval; CKD: chronic kidney disease; SUA: serum uric acid; CrCl: creatinine clearance.

For patients with and without tophi, 80% and 70%, respectively, of respondents reported considering a SUA level lower than 6 mg/dl as a target for ULT. Approximately 80% of respondents reported checking SUA every one to three months before the laboratory goal is achieved, and around 70% reported to verify it every four to six months after achieving the target. These answers are concordant with the ACR guidelines.

Over 42% of rheumatologists start allopurinol at doses of 300 mg daily for patients with normal renal function, which is in disagreement with the ACR guidelines. ULT was reported to be maintained indefinitely for patients without identified tophi only by 39.6% of respondents, whereas about one third reported maintaining ULT for less than one year after achieving the target SUA level.

Multivariate final models evaluating predisposing factors to answers about ULT in concordance to ACR guidelines are shown in Table 6. For two out of four questions, rheumatologists who had not attended a Rheumatology residency program were less likely to be in agreement to the ACR guidelines. Older physicians, those with more than 10 years of practicing rheumatology or time since graduation from medical school showed a reduced chance of concordance to the guidelines in at least one question. Chance of agreement was reduced for all regions compared to the South, but the difference failed to show statistical significance, except for the Midwest region for which the p-value reached a borderline level. Finally, not attending the 2013 BSR Meeting or any gout conference in that Meeting was associated with a reduced chance of being adherent to ACR recommendations.

**Table 6:** Multivariate-adjusted predictors of concordance between reported urate-lowering therapy management and the 2012 ACR gout guidelines.

Outcomes [Pseudo R2]	OR (CI 95%)	P
**Target serum uric acid level (<5 or <6 mg/dl) for patients with tophi [0.0713]**		
a) Age (continuous variable in years)	0.97 (0.95–0.99)	**0.015**
b) Residency in rheumatology (ref.: yes)	0.56 (0.30–1.03)	0.062
c) Attendance at the 2013 BSR Meeting (ref.: yes)	0.45 (0.25–0.80)	**0.007**
**Target serum uric acid level (<5 or <6 mg/dl) for patients without tophi [0.0376]**		
a) Time practicing Rheumatology (ref.: 0–10 years)		
11–20 years	0.44 (0.22–0.86)	**0.018**
21–30 years	0.58 (0.28–1.20)	0.142
≥31 years	0.45 (0.21–0.98)	**0.043**
b) Attendance at any gout lecture at the 2013 BSR Meeting (ref.: yes)	0.46 (0.25–0.84)	**0.011**
**Initial dose of allopurinol (50 or 100 mg/day) for patients with normal renal function [–]**		
None	**–**	**–**
**Initial dose of allopurinol (50 or 100 mg/day) for patients with CrCl ≤60 ml/min [0.0920]**		
a) Time since Medical School graduation (ref.: 0–10 years)		
11–20 years	0.32 (0.06–1.67)	0.178
21–30 years	0.22 (0.04–1.14)	0.072
≥31 years	0.25 (0.05–1.26)	0.093
b) Geographic region of Brazil (ref.: South)		
Midwest	0.11 (0.01–1.10)	0.060
North	0.13 (0.01–1.66)	0.117
Northeast	0.21 (0.02–1.88)	0.161
Southeast	0.23 (0.03–1.81)	0.162
c) Residency in rheumatology (ref.: yes)	0.36 (0.15–0.85)	**0.019**

ACR: American College of Rheumatology; OR: odds ratio; CI: confidence interval; BSR: Brazilian Society of Rheumatology; CrCl: creatinine clearance. OR lower than 1 represents a reduced chance of concordance with the 2012 ACR gout guidelines.

## Discussion

This is the first representative national physician survey assessing adherence to the 2012 ACR gout guidelines. We used a random sample of Brazilian rheumatologists to perform this survey and had a 78% response rate. We noted discordance between practice and the ACR gout guidelines related to the use of colchicine in the treatment of an acute gout attack, ULT discontinuation during an acute gout attack, duration of anti-inflammatory prophylaxis, initial high-dose and duration of ULT. We found practice was concordant with ACR gout guidelines with regards to the treatment of acute gout attack, prescription of anti-inflammatory prophylaxis when initiating ULT, SUA monitoring and target SUA achievement. Several findings merit further discussion.

We observed problems in acute gout management that reflected discordance between guidelines and practice. First, practitioners reported the use of colchicine in patients with acute gout >36 hours duration, which is not recommended by the guidelines. This is based on pharmacokinetics and clinical observations that colchicine should preferably be started early in an acute attack and for this clinical scenario, other treatment options, such as corticosteroids and NSAIDs are more suitable [23]. Another observation was the use of high-dose colchicine of >2 mg/day. This is again a safety issue, given that a recent randomized trial showed equal efficacy with <2 mg/day compared to >2 mg/day dose and higher toxicity with the higher dose [47]. We recognize that the rates of discordance were low, but it is possible that many other rheumatologists consider these therapeutic approaches in their daily practice, since the question was restricted to the first choice treatment in predetermined situations. Both prescriptions represent a higher risk to benefit for colchicine and given a clear guidance from the ACR guidelines, we think this needs to be corrected.

We observed a discordance in the management of anti-inflammatory prophylaxis between the ACR guidelines and physician practice, with some reporting continuing prophylaxis indefinitely after initiating ULT while others prescribed anti-inflammatory prophylaxis for <1 month. Thus, the duration of anti-inflammatory prophylaxis was either too long or too short for some gout patients starting ULT. A short duration of prophylaxis may lead to frequent gout flares during ULT dose adjustment and an indefinite duration, without a clear indication, put the patient on an unfavorable risk-benefit balance [48]. This identifies another area where physician education might help to improve practice.

Withdrawing ULT during an acute gout attack was reported by a higher than expected proportion of respondents, which is in disagreement with the current guidelines. Stopping ULT during an acute attack may lead to persistence or a more difficult to control gout attack [14].

The maintenance of ULT indefinitely was only reported as advocated by 76.3% and 39.6% of respondents for patients with and without tophi, respectively, contrary to the ACR guidelines recommendation of continuing indefinitely all measures needed to maintain SUA lower than 6 mg/dl.

Only half of respondents reported starting allopurinol at doses of 100 mg daily or less for patients with normal renal function, as recommended by the ACR guidelines, while 42.7% reported to prescribe an initial dose of 300 mg daily. The prescription of 300 mg/day starting dose of allopurinol is inconsistent with the current guidelines, and this higher dose has associated potential higher risk of allopurinol hypersensitivity reaction as well as more gout flares [14,49].

Based on the results of the multivariate analysis, we conclude that the recent changes in gout management may not have been communicated effectively to a significant proportion of rheumatologists; rheumatologists who were older, those in practice for a longer time duration and those working exclusively in private practice had higher rates of discordance with the gout guidelines.

We observed several practice patterns for gout management that are concordant with ACR guidelines. It was reassuring to observe that anti-inflammatory prophylaxis was commonly instituted when initiating ULT (92.4%), with a percentage very similar to results from previous studies: 90% (US, 2006) [37] and 95% (France, 2013) [27]. We noted that a high percentage of rheumatologists aimed at a SUA target of lower than 6 mg/dl or 5 mg/dl in non-tophaceous and tophaceous gout, respectively. We considered both answers as concordant with ACR guidelines that recommend a SUA target of <6 mg/dl, but suggest reducing it below 5 mg/dl in some cases, including in the presence of tophus. Our findings are consistent with an earlier survey of US rheumatologists where 84% of respondents reported to aim for a SUA level lower than 6 mg/dl, but the question did not discriminate between patients with or without tophi, and the response rate was lower than to our survey [37]. Most respondents reported checking SUA every one to three months before achieving the SUA target and every four to six months after the target has been achieved. The ACR guidelines recommend monitoring of SUA every 2 to 5 weeks during ULT titration, and every 6 months once the SUA target is achieved, a measure believed to enhance patient compliance.

In our survey, in an otherwise healthy patient, corticosteroids (monotherapy or in combination with colchicine) were prescribed for acute gout by 7.8% respondents for monoarticular attack with <36 hours duration and by 30.1% for polyarticular attack with >36 hours duration. On the other hand, 90% of rheumatologists appropriately used corticosteroids in a polyarticular attack lasting more than 36 hours, in a patient with CKD. This higher utilization of corticosteroids in the presence of CKD likely indicates the appropriate avoidance of NSAIDs and possibly colchicine, due to known adverse events associated with their use in these populations [50,51]. The low rate of intraarticular corticosteroid injections for monoarticular attacks was surprising, but has been observed in other survey studies [31,34].

It is important to highlight study limitations. Non-response should be taken into account while interpreting findings, especially considering that more responders were board-certified and attended the 2013 BSR Meeting compared with non-responders, suggesting that results from this survey are likely to overestimate general rheumatologists' behavior. However, our response rate for a physician survey is two to three times greater than that of previous surveys [26–44] and well in excess of what is reported for physician surveys in general. Due to study design and limited resources, we did not audit patient charts for physicians responding to the survey, and therefore we were unable to confirm whether the practice reported by the physician is actually truly reflective of management of their gout patients. We believe that physicians reported their real practice, not what they considered as the expected or “correct” answer, as instructed explicitly in our survey. We do not think that these findings can be generalized to general medicine practice in Brazil or to other country settings. Such studies, in the future, will allow a better understanding of current gout management and how this can be improved. We recognize that rheumatologists are not the primary providers of gout care; however, they are believed to be the key opinion leaders in this topic. The main strength of our nation-wide study is the representativeness of our sample, which was randomly selected, a high participation rate (typically unusual for physician surveys) and comprehensive assessment of key ACR gout guidelines domains.

We have identified several areas that should be the focus of continued education on gout treatment for the Brazilian rheumatologists, based on this survey assessing the adherence to 2012 ACR guidelines for the management of gout [22,23]. These include: 1) not to start colchicine as monotherapy when an attack has lasted longer than 36 hours; 2) avoidance of high-dose colchicine; 3) maintenance of a stable dose of ULT during acute attacks; 4) duration of anti-inflammatory prophylaxis for at least six months; 5) duration of ULT; and 6) the need to initiate allopurinol in low doses.

## Conclusion

In conclusion, in this representative study of Brazilian rheumatologists, we found that several practice patterns were concordant with the 2012 ACR gout guidelines, but also that an opportunity exists to improve gout management in several areas. Several physician characteristics were associated with discordance with the ACR guidelines. Based on these data, we are developing a physician education program to fill these knowledge gaps and improve practice. Our aim is to improve the management of gout patients in Brazil using this program.

## Supporting Information

S1 File Questionnaire used in the survey. (PDF)

S1 Table Similar surveys. (DOCX)
