# Supplementary material for: Adherence to the 2012 American College of Rheumatology (ACR) Guidelines for Management of Gout: A Survey of Brazilian Rheumatologists
Source: PLoS One. 2015 Aug 14;10(8):e0135805. doi: 10.1371/journal.pone.0135805 (PMC4537114; doi:10.1371/journal.pone.0135805)
Supplement: S1 File — (PDF) [file pone.0135805.s001.pdf]

Survey of Gout Treatment in Brazil

THE GOUT COMMITTEE OF THE BRAZILIAN SOCIETY OF RHEUMATOLOGY NEEDS YOUR ASSISTANCE!

Dear colleagues,

We are conducting a survey on gout treatment practices by Brazilian Rheumatologists.

Responses should reflect clinical practice, with no concern for right or wrong.

The data will be evaluated without any identification of responders.

It will take less than 10 minutes!
Join!
Thank you!

Gout Committee

Sociedade Brasileira de Reumatologia
Comissão de Gota

About the pharmacological approach of acute gouty attack

1. What is your first choice for an acute gouty attack in an OTHERWISE HEALTHY PATIENT?

	colchicine 0.5 mg/hour until symptom resolution or side effect	colchicine ≤ 2 mg/day	Nonsteroidal anti-inflammatory drug (NSAID)	PO steroid	IM steroid	Intra-articular steroid	NSAID + colchicine	NSAID + steroid (PO/IM/IA)	Steroid (PO/IM/IA) + colchicine
Monoarticular involvement, onset < 36 hours	○	○	○	○	○	○	○	○	○
Monoarticular involvement, onset > 36 hours	○	○	○	○	○	○	○	○	○
Polyarticular involvement, onset < 36 hours	○	○	○	○	○	○	○	○	○
Polyarticular involvement, onset > 36 hours	○	○	○	○	○	○	○	○	○

2. What is your first choice for an acute gouty attack in a patient with CREATININE CLEARANCE (CrCl) ≤ 60 ML/MIN?

	colchicine 0.5 mg/hour until symptom resolution or side effect	colchicine ≤ 2 mg/day	Nonsteroidal anti-inflammatory drug (NSAID)	PO steroid	IM steroid	Intra-articular steroid	NSAID + colchicine	NSAID + steroid (PO/IM/IA)	Steroid (PO/IM/IA) + colchicine
Monoarticular involvement, onset < 36 hours	○	○	○	○	○	○	○	○	○
Monoarticular involvement, onset > 36 hours	○	○	○	○	○	○	○	○	○
Polyarticular involvement, onset < 36 hours	○	○	○	○	○	○	○	○	○
Polyarticular involvement, onset > 36 hours	○	○	○	○	○	○	○	○	○

3. During an acute gouty attack in a patient using urate-lowering therapy (ULT) (eg. allopurinol), you:

- ○ Increase the dose of the ULT
- ○ Keep the dose of the ULT
- ○ Reduce the dose of the ULT
- ○ Withdraw the ULT

Survey of Gout Treatment in Brazil

About urate-lowering therapy (ULT)

4. When would you initiate ULT? Check all that apply.

- ☐ After their first gouty attack.
- ☐ After two or more gouty attacks a year.
- ☐ When a patient with gout has tophi.
- ☐ When a patient with gout has chronic kidney disease.
- ☐ None of the above.

5. Choose the serum uric acid (SUA) level you consider the goal for ULT for a patient WITH TOPHI:

- ○ < upper limit of normal
- ○ < 6.8 mg/dl
- ○ < 6.0 mg/dl
- ○ < 5.0 mg/dl
- ○ I do not titrate ULT based on the SUA level

Questions 6 to 10 are only offered to those who did not answer "I do not titrate ULT based on the SUA level" in the question 5.

6. Choose the SUA level you consider the goal for ULT for a patient WITHOUT TOPHI:

- ○ < upper limit of normal
- ○ < 6.8 mg/dl
- ○ < 6.0 mg/dl
- ○ < 5.0 mg/dl
- ○ I do not titrate ULT based on the SUA level

7. How often do your patients reach this target with current treatments?

- ○ Always
- ○ Almost always
- ○ Sometimes
- ○ Almost never
- ○ Never

8. For how long after achieving the target SUA level do you prescribe ULT for a patient with gout WITHOUT tophi?

- ○ Withdraw the medication
- ○ Maintain for 1 - 6 months
- ○ Maintain for 7 - 11 months
- ○ Maintain for 1 - 3 years
- ○ Maintain for 4 or more years
- ○ Maintain indefinitely

About urate-lowering therapy (ULT)

9. For how long after achieving the target SUA level do you prescribe ULT for a patient with gout WITH tophi?

- Withdraw the medication
- Maintain for 1 - 6 months
- Maintain for 7 - 11 months
- Maintain for 1 - 3 years
- Maintain for 4 or more years
- Maintain until tophi resolution
- Maintain indefinitely

10. How often do you check your patients SUA levels?

	1-3 months	4-6 months	7-9 months	10-12 months	I do not check it.
Before achieving the target SUA level:	○	○	○	○	○
After achieving the target SUA level:	○	○	○	○	○

11. How many of your patients have ever presented an allergic reaction to allopurinol under your care?

	0	1 - 5	6 - 10	11 - 15	> 15
Mild reaction	○	○	○	○	○
Severe reaction	○	○	○	○	○

12. In general, when you initiate allopurinol, what is the initial dose you prescribe?

	50 mg/day	100 mg/day	200 mg/day	300 mg/day
For a patient with normal renal function	○	○	○	○
For a patient with CrCl ≤ 60 ml/min	○	○	○	○

13. What is the maximum dose of allopurinol most frequently prescribed by you?

	≤ 300 mg/day	350 - 450 mg/day	500 - 600 mg/day	650 - 750 mg/day	800 - 900 mg/day
For a patient with normal renal function	○	○	○	○	○
For a patient with CrCl ≤ 60ml/min	○	○	○	○	○

About urate-lowering therapy (ULT)

14. Do you prescribe BENZBROMARONE?

	always	almost always	sometimes	almost never	never
For patients with CrCl > 60 ml/min	○	○	○	○	○
For patients with CrCl between 30 and 60 ml/min	○	○	○	○	○
For patients with CrCl < 30 ml/min	○	○	○	○	○
For patients with current renal underexcretion of uric acid and a history of kidney stones in the past	○	○	○	○	○
For patients also using ALLOPURINOL	○	○	○	○	○

15. If you were to initiate ULT in a patient, would you wait for the resolution of the acute gouty attack?

○ Yes

○ No

16. How long after resolution of the acute gouty attack would you wait to initiate ULT?

○ 1 - 3 weeks

○ 4 - 6 weeks

○ 7 - 9 weeks

○ 10 - 12 weeks

This question is offered only to those who answered "YES" to the question 15.

About prophylaxis of acute gouty attacks

17. How often do you give prophylatic treatment to prevent acute gouty attacks when initiating ULT?

- Always
- Almost always
- Sometimes
- Almost never
- Never

The following questions in this page are not offered to those who answered "NEVER" to the question 17.

18. How long do you keep prophylaxis for patients WITHOUT tophi?

- < 1 month
- 1 - 6 months
- 7 - 12 months
- Until they reach the target SUA level
- Indefinitely

19. How long do you keep prophylaxis for patients WITH tophi?

- < 1 month
- 1 - 6 months
- 7 - 12 months
- Until they reach the target SUA level
- Until resolution of tophi
- Indefinitely

20. Do you prefer colchicine or NSAID for chronic prophylaxis of acute gouty attacks?

- Colchicine
- NSAID

About you

21. Gender:

- Female
- Male

22. About you

	Age	Time since Medical School graduation	Time practicing Rheumatology
In years			

23. State in which you practice:

State	

24. On average, number of gout patients you see monthly:

Patients with gout/month	

25. Type of practice

- Private
- Academic
- Combined

26. Residency or specialization in Rheumatology?

- No
- Residency
- Specialization

27. Did you attend the Brazilian Society of Rheumatology Meeting in 2013 (2013 BSR Meeting)?

- Yes
- No

Survey of Gout Treatment in Brazil

About you

28. Have you been to a gout lecture at 2013 BSR Meeting?

- ○ Yes
- ○ No

This question is offered only to those who answered "YES" to the question 27.

Thank you very much for your participation!

Sociedade Brasileira de Reumatologia
Comissão de Gota
