# Supplementary material for: Adherence to the 2012 American College of Rheumatology (ACR) Guidelines for Management of Gout: A Survey of Brazilian Rheumatologists
Source: PLoS One. 2015 Aug 14;10(8):e0135805. doi: 10.1371/journal.pone.0135805 (PMC4537114; doi:10.1371/journal.pone.0135805)
Supplement: S1 Table — (DOCX) [file pone.0135805.s002.docx]

**S1 Table: Similar surveys.**

| **Place,**  **year of publication** | **Source population** | **Selected for contact** | **Responses** | **Overall response rate** |
| --- | --- | --- | --- | --- |
| Austria, 2014 [26] | Members of the Austrian Society of Rheumatology | WSP (574) | 127 | 22.1% |
| French Polynesia, 2014 [27] | Rheumatologists and GPs from the French Polynesia | N/A | 49 | N/A |
| USA, 2013 [28] | Primary Care Practitioners from the American Medical Association | Random nationwide sample of 2200 PCPs | 838 | 40.1%^1^ |
| France, 2013 [29] | Rheumatologists and GPs from the France | N/A | 977 | N/A |
| Argentina, 2012 [30] | Rheumatologists, internists and GPs from Buenos Aires city | Professionals from different scenarios^2^ | 171 | ≤ 20%^3^ |
| Malaysia, 2009 [31] | Doctors attending rheumatology post-graduate courses | Doctors attending courses in 2005 where the authors were invited speakers | 145 | 54.5% |
| USA, 2008 [32] | Primary Care Practitioners from the USA | PCPs attending a series of 25 accredited educational meetings from April through September 2006 | 688 | N/A |
| Dutch, 2008 [33] | Dutch rheumatologists and those in training, members of the Dutch Society of Rheumatology | WSP (252) | 122 | 50.4% |
| Ireland, 2008 [34] | GPs in the North Dublin GP Partnership | WSP (170) | 80 | 47.0% |
| Eular, 2007 [35] | Delegates attending the Eular Meeting – 2006 | Delegates visiting the commercial stands | 741 | 6.7%^4^ |
| China, 2006 [36] | Internists from PUMC Hospital, Beijing | Physicians attending medical and rheumatology grand rounds (121) | 93 | 76.9% |
| USA, 2006 [37] | Rheumatologists from the USA | The first 2500 American rheumatologists listed alphabetically in the 2004-2005 ACR directory with fax numbers | 518 | 20.7% |
| China, 2006 [38] | Physicians from China | 121 internists from PUMC Hospital [36], and 75 physicians attending a national CME workshop of rheumatology | 100 | 51.0% |
| Mexico, 2003 [39] | Rheumatologists, internists, orthopedic surgeons and GPs from Mexico City | All rheumatologists (133) and internists (423); and a random sample of orthopedic surgeons (640) and GPs (640) | 212 | 11.5% |
| France, 1996 [40] | French rheumatologists | 2520 French rheumatologists | 750 | 29.8% |
| Brazil, 1994 [41] | Rheumatologists and GPs from the city of São Paulo | All rheumatologists (252) and a random sample of GPs (500) | 421 | 56.0% |
| New Zealand, 1991 [42] | Rheumatologists and GPs from New Zealand | All rheumatologists (27) and a 10% random sample of GPs (207) | 189 | 80.8% |
| Australia, 1989 [43] | Rheumatologists and GPs from New South Wales and Queensland | All rheumatologists (85) and a random sample of GPs (430) | 326 | 63.3% |
| Canada,1988 [44] | Rheumatologists and GPs from the Province of Ontario | All rheumatologists (87) and a random sample of GPs (200) | 189 | 65.8% |

^1^After excluding physicians who reported not caring for gout patients (n=51) or those not contacted due to incorrect addresses (n=61); ^2^professionals assisting to different scientiﬁc events related to rheumatology, physicians working in several emergency rooms and medical clinics, and members of the Rheumatology department of the Hospital JM Ramos Mejia; ^3^believed by the authors to be ≤ 20%; ^4^ percentage of attendees at the Eular Meeting 2006. WSP: whole source population; GPs: general practitioners; N/A: not available; PCPs: primary care physicians; PUMC Hospital: Peking Union Medical College Hospital; Eular: European League Against Rheumatism; ACR: American College of Rheumatology; CME: continuous medical education.
